# Long‐acting injectable paliperidone palmitate for severe anorexia nervosa and comorbid autism spectrum disorder: A case report

**DOI:** 10.1002/pcn5.70161

**Published:** 2025-07-13

**Authors:** Yuhei Mori, Yuhei Suzuki, Itaru Miura

**Affiliations:** ^1^ Department of Neuropsychiatry School of Medicine, Fukushima Medical University Fukushima Japan

**Keywords:** anorexia nervosa, autism spectrum disorder, long‐acting injectable antipsychotics, paliperidone palmitate, treatment adherence

## Abstract

**Background:**

Anorexia nervosa (AN), comorbid with autism spectrum disorder (ASD), poses significant treatment challenges due to cognitive rigidity, poor insight, and frequent nonadherence to pharmacological interventions. Although second‐generation antipsychotics (SGAs) have been used off‐label in AN, evidence for long‐acting injectable (LAI) formulations remains scarce, particularly in adult patients with neurodevelopmental disorders.

**Case Presentation:**

We report the case of a 27‐year‐old woman with severe AN and comorbid ASD who exhibited repeated hospitalizations due to critical underweight and persistent refusal of oral medications. Cognitive assessment revealed mild intellectual disability (IQ 56). The patient demonstrated obsessive‐compulsive traits and extreme rigidity toward food intake, and was resistant to multiple oral antipsychotics. While risperidone was tolerated, poor adherence limited its efficacy. After obtaining informed consent, LAI paliperidone palmitate was initiated (initial dose 25 mg, increased to 50 mg monthly). Following a short period of psychoeducation and lifestyle intervention, the patient maintained psychiatric and nutritional stability over a 3‐year outpatient follow‐up without rehospitalization. Her body mass index stabilized at approximately 24 kg/m^2^. No severe adverse effects were reported.

**Conclusion:**

This case highlights the potential role of LAI paliperidone palmitate in managing treatment‐refractory AN with comorbid ASD and intellectual disability, particularly in patients with poor adherence and prominent obsessive traits. Although antipsychotics are not standard for AN, LAI formulations may offer pragmatic, sustainable benefits in selected cases. Further studies are warranted to assess long‐term safety and efficacy in this population.

## BACKGROUND

Anorexia nervosa (AN) is a severe psychiatric disorder associated with high morbidity and mortality. Treatment resistance is common, especially in individuals with comorbid neurodevelopmental disorders such as autism spectrum disorder (ASD), who frequently exhibit cognitive rigidity, obsessive‐compulsive traits, and poor adherence to pharmacological interventions. In addition, core ASD traits such as insistence on sameness, cognitive inflexibility, and social withdrawal may contribute to poor engagement with standard psychotherapeutic or nutritional approaches in AN. Although atypical antipsychotics have been explored for AN,^1^, ^2^ evidence supporting the use of long‐acting injectable (LAI) formulations in this population remains limited. A recent case report by Moradi et al. described the successful short‐term use of LAI risperidone in an adolescent with AN and ASD, reporting improvements in weight and outpatient stability.^3^ Herein, we report the successful intermediate‐term use of monthly paliperidone palmitate in a young adult with severe AN and comorbid ASD.

The patient and her family provided consent for the publication of this case. This report obeyed the rule of the Ethics Committee of Fukushima Medical University Hospital and conformed to the provisions of the Declaration of Helsinki.

## CASE PRESENTATION

We describe the case of a 27‐year‐old woman with severe AN and comorbid ASD who was hospitalized due to critically low body weight and persistent refusal of oral medications. The patient had exhibited early signs of social and interpersonal difficulties, including restricted interests and impaired communication throughout childhood; however, no overt language delay was noted. The patient graduated from a general high school but encountered significant challenges in the workplace due to social communication difficulties, ultimately leading to early job termination. Subsequent psychiatric evaluation led to a diagnosis of ASD. Cognitive assessment using the Wechsler Adult Intelligence Scale – Third Edition revealed a full‐scale IQ of 56, consistent with mild intellectual disability. The patient remained at home for approximately 3 years, during which time her dietary restrictions progressively worsened.

At age 27, the patient was admitted for the first time with severe underweight (body mass index [BMI] approximately 12 kg/m^2^) and developed clinical signs of refeeding syndrome. This initial hospitalization lasted for approximately 6 months, during which her weight increased to a BMI of approximately 16 kg/m^2^. During this period, the patient exhibited marked obsessive behaviors and cognitive rigidity regarding food intake, consistent with ASD‐related traits.

During the second hospitalization, oral risperidone, asenapine, and aripiprazole were sequentially introduced to target her restrictive and rigid eating behaviors. Asenapine and aripiprazole were discontinued due to intolerable akathisia. Although the patient largely accepted oral risperidone during hospitalization, her limited insight into her illness raised concerns regarding medication adherence after discharge. While the patient did not meet formal diagnostic criteria for a psychotic disorder, she demonstrated severe behavioral and emotional dysregulation related to feeding behavior, unresponsive to standard interventions, which appeared to contribute to her persistent low body weight. Among antipsychotics approved for use in individuals with ASD, risperidone and aripiprazole are commonly considered; however, the latter had previously been poorly tolerated due to akathisia. After thorough discussion within a multidisciplinary team and obtaining informed consent from both the patient and her family, we judged the use of LAI paliperidone to be ethically justifiable in order to achieve behavioral stabilization and support long‐term management. LAI antipsychotic treatment with paliperidone palmitate was initiated 2 weeks before discharge. As the LAI formulation of risperidone requires biweekly administration, it was considered potentially burdensome for sustained adherence in this patient. Given that paliperidone is the major active metabolite of risperidone and is available as a once‐monthly LAI, we selected paliperidone palmitate as a more practical and sustainable alternative.

**Figure 1:**
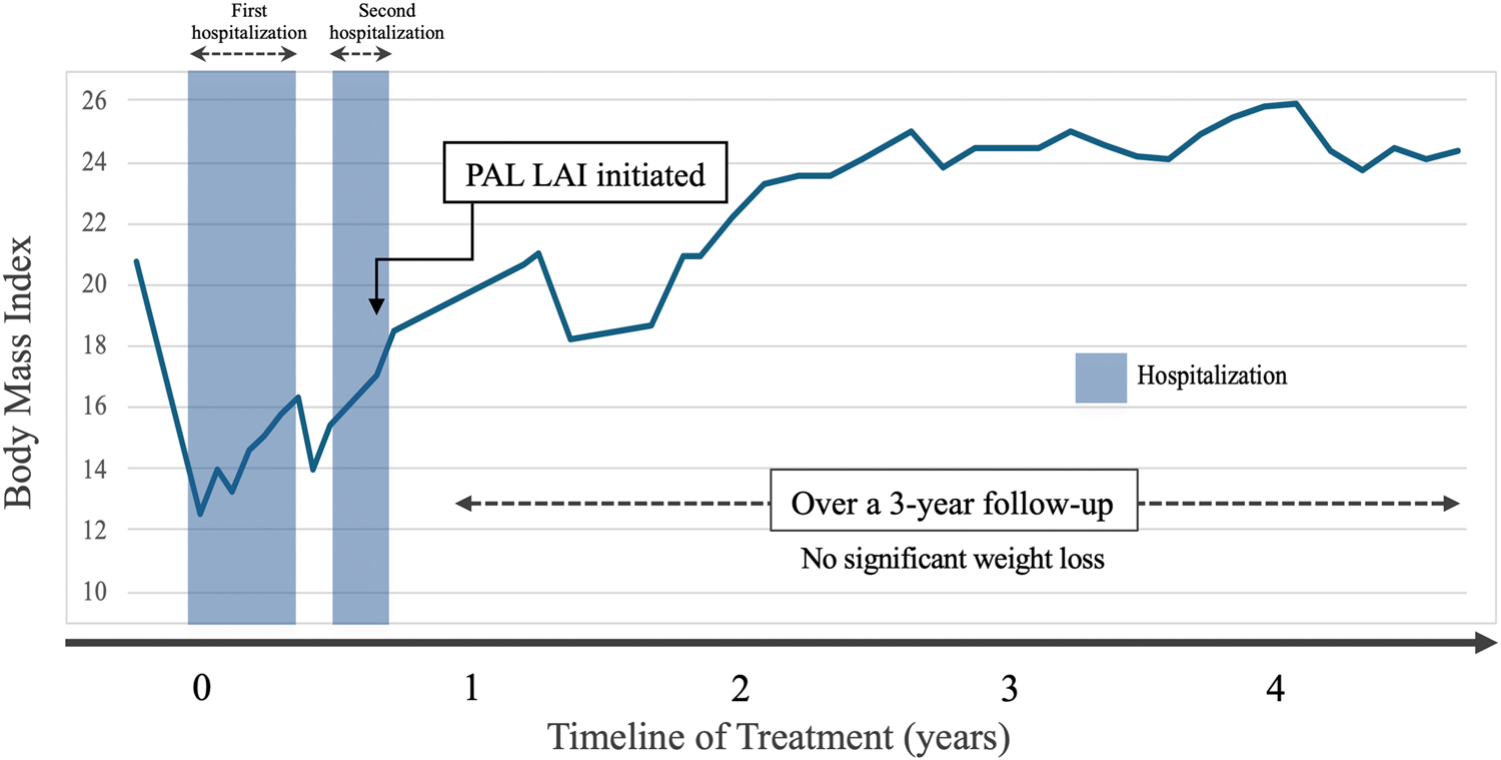
Weight trajectory before and after paliperidone long‐acting injectable (LAI) initiation. The figure illustrates the patient's weight changes from the first hospitalization to the outpatient follow‐up period. During the initial admission, nutritional rehabilitation led to a gradual increase in body mass index (BMI). Following discharge and subsequent readmission, oral antipsychotics including risperidone were introduced, but adherence concerns persisted. LAI paliperidone palmitate (monthly formulation) was initiated during the second hospitalization after the patient reached a BMI over 18. After LAI initiation, sustained weight stabilization and outpatient maintenance were observed for over 3 years. PAL LAI, paliperidone palmitate LAI.

The formulation used was the monthly preparation of paliperidone palmitate. The initial dose was 25 mg, increased to 50 mg after 2 months, which was maintained thereafter. Given the potential for adverse effects such as extrapyramidal symptoms and cardiovascular complications in severely underweight patients, we considered it prudent to initiate LAI antipsychotic treatment only after the patient had achieved a BMI > 18 kg/m^2^. This decision was based on the clinical judgment that a certain level of physical recovery was necessary to mitigate the risk of pharmacological side effects. Following the initial administration of LAI paliperidone palmitate, the patient underwent a 2‐month voluntary hospitalization focused on psychoeducation and lifestyle interventions. Since then, the patient has remained psychiatrically stable in the outpatient setting for over 3 years. The body weight has stabilized at approximately 56.2 kg, with fluctuations of ±1 kg (Figure 1). Although some obsessive thoughts about food persist, the patient has exhibited no significant restrictive eating behaviors and has not required rehospitalization.

## DISCUSSION

This case highlights the potential utility of LAI paliperidone palmitate in a 27‐year‐old woman with AN and comorbid ASD who presented with poor medication adherence, severe food refusal, and critical underweight requiring hospitalization. While antipsychotics are not standard treatments for AN, their off‐label use has been explored in cases characterized by comorbid ASD, prominent obsessive‐compulsive features, affective instability, and cognitive rigidity. A meta‐analysis by Dold et al. reported that second‐generation antipsychotics (SGAs) may offer modest benefits in AN; however, the overall quality of evidence was low, and no studies specifically examined LAI formulations or ASD subgroups.^4^ A more recent scoping review by Thorey et al. further noted that although SGAs are commonly prescribed in clinical practice for AN, robust efficacy data for risperidone and paliperidone remain limited.^5^

In the present case, the patient had previously tolerated oral risperidone but experienced intolerable akathisia with asenapine and aripiprazole. Given her anticipated nonadherence to oral medications and the need for sustained stabilization, LAI paliperidone palmitate was selected. Compared to risperidone, LAI paliperidone offers several pharmacokinetic advantages, including more stable plasma concentrations, reduced hepatic metabolism, and a longer dosing interval (monthly vs. biweekly), which may be better suited to patients with ASD‐related behavioral rigidity and aversion to frequent medical interventions. Although a 3‐month formulation of LAI paliperidone is available, it had not yet been introduced at our hospital at the time of this case, therefore we opted for the once‐monthly formulation as a more feasible option.

Simpson et al. demonstrated the safety and efficacy of LAI paliperidone in managing irritability and behavioral dysregulation in pediatric patients with ASD and intellectual disability.^6^ In our case, the decision to initiate antipsychotic treatment was driven by the presence of food‐related obsessions and compulsive behaviors, reflecting cognitive inflexibility associated with ASD rather than primary psychosis. Notably, we initiated LAI treatment only after the patient's BMI had recovered above 18, based on clinical judgment that a minimum level of physical restoration was necessary to mitigate the risk of extrapyramidal and cardiovascular side effects, which are more likely in underweight individuals.

Following LAI initiation, the patient achieved full weight restoration (BMI approximately 24) and maintained both nutritional and psychiatric stability for over three years without requiring rehospitalization. Although some preoccupations with food persisted, restrictive eating behaviors did not recur. The patient's clinical presentation did not meet the criteria for schizophrenia as defined in the Diagnostic and Statistical Manual of Mental Disorders, Fifth Edition. Her case is best conceptualized as severe AN complicated by neurodevelopmental vulnerabilities, including ASD and mild intellectual disability.

This case complements a recent report by Moradi et al., which described short‐term stabilization with LAI risperidone in an 11‐year‐old girl with AN and ASD.^3^ Compared to the previous case report by Moradi et al., which described short‐term improvement in an 11‐year‐old adolescent with AN and ASD treated with biweekly risperidone LAI, our case presents several distinct and clinically meaningful differences. First, our patient was a young adult with more severe and complex comorbidities, including ASD, intellectual disability, and prior intolerance to multiple oral antipsychotics. Second, we observed behavioral stabilization and weight restoration over a longer treatment course, with sustained outpatient follow‐up. Third, we employed a once‐monthly LAI formulation (paliperidone palmitate), which may reduce treatment burden and enhance adherence, especially in individuals with poor insight and limited tolerance to daily medication. These factors collectively contribute novel and practical insights to the limited body of literature on LAI antipsychotic use in this unique clinical population.

Moreover, growing evidence indicates that individuals with elevated autistic traits often experience lower engagement in conventional eating disorder (ED) treatments and higher levels of treatment resistance. A mixed‐method systematic review by Nimbley et al. found that patients with ASD or high autistic traits frequently perceive ED treatment environments as overwhelming, leading to lower treatment satisfaction and increased rates of inpatient care.^7^ Similarly, Zhang et al. reported that both an ASD diagnosis and elevated polygenic risk scores for autism were associated with greater ED symptom severity and poorer treatment response.^8^ These findings underscore the importance of individualized, neurodevelopmentally informed treatment strategies for patients with AN and co‐occurring ASD traits. In our case, the patient demonstrated sustained improvement only after receiving pharmacologic intervention tailored to her cognitive and behavioral profile.

Nevertheless, further prospective studies are warranted to assess the generalizability, safety, and long‐term outcomes of LAI antipsychotic treatment in individuals with AN, particularly those with comorbid neurodevelopmental conditions.

## CONCLUSION

This case demonstrates the potential utility of LAI paliperidone palmitate as a long‐term treatment strategy for patients with severe AN and comorbid ASD, particularly when medication adherence is a concern. Tailoring antipsychotic interventions to neurodevelopmental profiles may enhance treatment engagement and facilitate sustained psychiatric and nutritional stabilization. Further research is needed to establish the broader applicability of this approach.

## AUTHOR CONTRIBUTIONS

Yuhei Mori conceptualized and designed the study, provided clinical care for the patient, contributed to data interpretation, and was responsible for drafting the manuscript and creating the figure. Yuhei Suzuki and Itaru Miura supervised the work. All authors have reviewed the manuscript and approved its submission.

## CONFLICT OF INTEREST STATEMENT

The authors declare no conflicts of interest.

## ETHICS APPROVAL STATEMENT

The Committee of Fukushima Medical University approved this study.

## PATIENT CONSENT STATEMENT

The patient provided written informed consent for the publication of this report.

## CLINICAL TRIAL REGISTRATION

N/A.

## Data Availability

Data sharing not applicable to this article as no datasets were generated or analysed during the current study.
